# An Analysis of the COVID-19 Situation in India in Terms of Testing, Treatment, Vaccine Acceptance and National Economic Performance

**DOI:** 10.3389/ijph.2022.1604975

**Published:** 2022-11-02

**Authors:** Ritu Bhaumik Patel, Bhaumik Bipinchandra Patel

**Affiliations:** ^1^ Department of Bioscience, Indrashil University, Rajpur, India; ^2^ Department of General Surgery, GMERS Medical College and Civil Hospital, Gandhinagar, India

**Keywords:** COVID-19 in India, COVID-19 testing, Coronavirus Vaccine, Economic impact of coronavirus, Indian healthcare system, COVID-19 statistics of India

## Abstract

**Objectives:** This study aims to provide a comprehensive review on the analysis of COVID-19 pandemic in India and address economic impact, diagnosis approaches, and vaccine acceptance and hesitation.

**Method:** We retrieved articles published in 2020 and 2021 and current data from official websites that narrate the strategy for COVID-19 testing, issues, and challenges, healthcare system insufficiency, statistics of cases, deaths, vaccination, and vaccine acceptance barriers, and beliefs.

**Results:** India being the 2nd largest populated country with a population of 1.4 billion faced massive difficulty in controlling the transmission of SARS-CoV-2. This crisis dramatically impeded the economy of the nation. India witnessed 2nd highest number (43,019,453) of confirmed cases and 3rd highest number of deaths (521,004) across the world.

**Conclusion:** The major cause of the collapse of COVID-19 is the high population of India, pre-existing weak healthcare system, and the lack of awareness among the people. The fall, rise, and statistics provided in the review will help in comparing the current status with other countries and in making strong strategies to combat future calamities.

## Introduction

The COVID-19 pandemic in India developed due to the global contagion of novel Coronavirus disease-2019 caused by the severe acute respiratory syndrome Coronavirus-2 (SARS-CoV-2). By 27th March 2022, India reported the second highest number (43,019,453) of confirmed Coronavirus cases across the globe and the third highest number (521,004) of deaths due to COVID-19 [1] (Table 1). The first case in India was reported in Thrissur, Kerala on 30th January 2020 in students returning from Wuhan, China [2]. Subsequently, 24th March 2020, witnessed a total of 9 deaths with 519 confirmed cases [3]. Owing to the imminent threat, the government announced a 21-day strict country-wide lockdown on 25th March 2020 [4]. Due to the severity of the situation, the National Disaster Management Authority further extended the lockdown to 31st May [5]. Meanwhile, since no standard treatment regime existed against COVID-19, different treatment modalities and preventive measures were tried to manage the condition of infected patients. In light of scientific and epidemiological evidence and less clarity on a treatment regime, vaccination was considered the primary focus and was aggressively pursued.

**TABLE 1:** COVID-19 dashboard of India by 27th March 2022 [1, 24] (India, March 2022).

Total samples tested	78,69,22,965
Total positive cases	4,30,19,453
New samples tested	6,20,251
New positive cases	1421
New positivity rate	0.23%
Total active cases	16,187
Total deaths	5,21,004
Total recovered cases	4,24,82,262
Total doses administered	1,830,285,290
People vaccinated 1st dose	1,003,924,602
People vaccinated 2nd dose	826,360,688

In January 2021, India approved Oxford–AstraZeneca (Covishield) vaccine, manufactured by the Serum Institute of India, Pune, and the first indigenous vaccine, BBV152 (Covaxin), developed by the National Institute of Virology, Pune, and manufactured by Bharat Biotech, Hyderabad [6]. On 16th January, almost a year after the first reported case in the country, a massive, free vaccination program was launched through 3,006 centers across the nation with the availability of Covishield or Covaxin, and approximately 165,714 people were vaccinated on the very first day [7]. The positivity rates kept on increasing till 10th June and then onwards recoveries exceeded active cases for the first time [8]. The deadly second wave of COVID-19 hit India in March 2021 and was more catastrophic than the first wave. The issue got compounded by the scarcity of hospital beds, vaccines, oxygen supply, and other medical services across all Indian states [9]. The public health system in India struggles on many fronts. High population density and poor socio-economic conditions in India further added to the struggle against the second wave [10, 11]. There was a sharp rise in the daily confirmed cases rate, surging from 1.62% on 1st March 2021 to 20% on 31st May 2021 [12]. The available resources (oxygen, drugs, ventilators, etc.) got exhausted quickly and hospitals had no isolation wards left, thereby leading to a massive number of casualties [13, 14]. Furthermore, the virus kept on mutating into more evolved strains that had more infectivity (higher reproduction number, R_0_) and could infect a wide range of age groups [15].

The most threatening strains detected in the country were United Kingdom variant: B.1.1.7, Brazilian variant: P.1, South African: B.1.351, and double-mutant Indian variant B.1.617 [16, 17]. The double-mutant Indian variant was extremely infectious and believed to have contributed to the uncontrollable surge in the second wave. The World Health Organization also declared this mutant as a “variant of global concern” [18]. On 30th April 2021, India became the first country to report active and new cases above 400,000 in a single day [19].

CODI-19 testing was instrumental in understanding how this pandemic is spreading and responding. It became the primary tool for identifying the infected population, monitoring treatment, isolation/quarantining infected individuals, and tracking down previous contacts. India implemented numerous approaches for screening and testing to prevent the transmission of the viruses. After the introduction of COVID-19 vaccines, some studies raised concerns such as safety, efficacy, efficiency, and negative aftereffects of the COVID-19 vaccines. Many rumors and realities surfaced about the necessity of vaccination, loopholes in the existing health system, and insufficient awareness of vaccine-treatable illnesses among the population [20–22]. This pandemic also proved to be a bane for the Indian economy. As per the Ministry of Statistics, the country’s economic growth declined from 6.1% in 2019 to 3.1% for the fiscal year 2020 in the 4th quarter [23].

Specifically, this study summarizes India’s response to COVID-19 in terms of adopting screening methods and its testing, treatment processes, and performance of the economy. The objectives of the study are to review the COVID-19 pandemic situation in India, a developing country, in terms of adopting screening methods and facing hurdles in testing and treatment, to summarize the effects of COVID-19 on the Indian economy, and to identify demographic and economic factors that affect the COVID-19 situation in the country.

## Methods

The COVID-19 data from 30th January 2020 to 27th March 2022 for India was retrieved from several official websites such as www.mygov.in/COVID-19 and www.mohfw.gov.in, important resources, i.e., ourworldindata.org/coronavirus and COVID19.who.int and reputed Indian newspapers such as Hindustan Times and Times of India. More than 50 research articles, reviews, mini-reviews, and reports have been cited that narrate the actual scenario on strategies and issues regarding COVID-19. This involves testing, approval, and temporal vaccination statistics, vaccination hesitation among population and gender, especially in women, and the social and economic impact of this pandemic. The collected data was analyzed and split into sections covering COVID-19 testing, vaccines and vaccination hesitation, and, economic impact. We summarized all the details of the COVID-19 pandemic surge and retraction in India for every sector as per changing timescale. A special focus has been given to the articles that offered quantitative evidence regarding the healthcare system response, clinical data of patients, and clinical manifestations related to COVID-19.

## Results

### COVID-19 Testing in India

A rapid and sensitive diagnosis of the COVID-19 virus proved to be critical for infection prognosis and pandemic control. As per the Indian Council of Medical Research (ICMR), Govt. of India report, a total of 26,49,72,022 cumulative samples were tested up to 16th April 2021 [24]. A total of 2,463 (1,233 government and 1,230 private) operational diagnostic laboratories were set up. Approximately 977/million individuals were tested daily and 13.20% of tests returned positive results showing high confirmed cases respective to the testing size, as WHO declared less than 5% positive rate is a good measure that infestation is under control [25]. Therefore, the rate of tests being carried out was not sufficient to monitor the pandemic properly and the actual number of infections could be far greater than the confirmed cases. As of 27th March 2022, India tested 78,69,22,965 samples with 4,30,19,453 confirmed cases [1] (Table 1). In the following sections, we discuss the tools and methods which have been adopted in India for COVID-19 testing.

#### Viral Antigen Detection

ICMR experimented with several plans to access the rate of transmission of infection, and out of them, one was a mass screening of suspected populations by rapid antigen test (RAT). As per the ICMR guidelines, RAT must be carried out before any confirmatory tests such as RT-PCR or CT scan for all the symptomatic and asymptomatic high-risk people residing in containment zones, hotspots, and entry points. As of 26th April 2021, a total of 95 rapid antigen test kits were checked by ICMR for having a minimal critical acceptance scale of ≥ 50% (for sensitivity) and ≥95% (for specificity), and only 35 kits were found to be competent and approved for diagnostic uses [24]. Undoubtedly, antigen tests could have been a visionary step or game-changer to contain this lethal pandemic within countries where the infection had initially spread. But the earlier tests were not highly sensitive as compared to the tests that rely on the detection of viral RNA such as RT-PCR which can detect as low as 100 copies per ml of viral RNA from a sample.

#### Viral Nucleic Acid Detection

**FIGURE 1:**
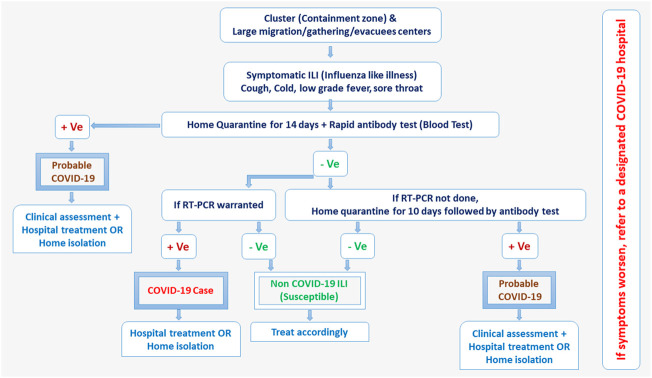
COVID-19 testing protocols released by government advisories of India (India, March 2022).

Real-time reverse transcription-polymerase chain reaction (RT-PCR) assays were employed most widely in India for the etiologic diagnosis of the COVID-19 infection and are considered the gold standard. Until 28th April 2021, ICMR evaluated 346 local and internationally available RT-PCR kits and out of these 346 kits, only 162 kits met the expectations [24]. ICMR recommended RT-PCR test for all symptomatic individuals who undertook international travel in the last 14 days, contacts of laboratory-confirmed cases, health care workers, and patients with severe acute respiratory illness. The asymptomatic direct and high-risk contacts of confirmed cases were recommended to be tested once between day 5 and day 14 of coming in his/her contact [24] (Figure 1). SARS-CoV-2 had been mutating and several variants were reported worldwide such as Brazilian (17 mutations), South African (12 mutations), and British (17 mutations), which could not be identified separately using RT-PCR testing. Therefore, whole-genome sequencing of random 13,614 samples was also performed and screened for these mutations. Of these, 1,109 samples were tested positive for British, 79 for South African, and one for Brazilian variant. Fortunately, the molecular testing being done in India did not miss these mutations and the specificity and sensitivity of RT-PCR assays remained consistent as earlier [26]. Private RT-PCR testing cost approximately 3,000 Indian rupees (INR) during the first wave of the pandemic and certainly, was unaffordable for common people in a developing country like India. To minimize the RT-PCR cost and resources being utilized, ICMR, in alignment with WHO recommended a practical approach of pooled sample testing in regions having disease incidences less than 5% [27].

#### Serology Testing

Antibody-based serological detection assays received significant interest as an alternative to nucleic acid testing for COVID-19 diagnosis. Serological tests are based on the quantification of SARS-CoV-2 specific immunoglobulin IgM, IgG, and IgA generated by human B-lymphocytes upon exposure to the virus. The advantage of immunological assays over the amplification assays was the ability to identify the previous infection history of individuals even though they never developed any symptoms. These assays helped healthcare professionals to produce data for those individuals who had overcome the disease and have generated an immune response against the pathogen. Consequently, this data aided in determining the contact tracing for the individuals who may donate convalescent plasma, a possible treatment for acute sufferers and patients who seek medical attention due to low immune system activity [28–30]. On 4th April 2020, ICMR issued recommendations to initiate antibody-based testing for SARS-CoV-2 in clusters and large gatherings in different parts of India.

#### CT-Scan

Lung computed tomography (CT) scan is a conventional and non-invasive viral detection tool that relies on X-rays transmission through the chest of the patient. In the case of COVID-19 diagnosis, perilobular pattern, reverse halo, air bronchograms, and consolidation were the potential characteristics in CT images that might provide 100% coverage. As per ICMR findings, rapid antigen testing had only 40% sensitivity and RT-PCR assays had approximately 80% sensitivity in India, leaving behind a 20% uncertainty for remaining cases which might still spread the infection [31]. So, regardless of radiation exposure, chest CT scanning was proposed as an option for diagnosing and monitoring COVID-19 infection in suspected cases where molecular testing failed.

### Vaccination in India

**FIGURE 2:**
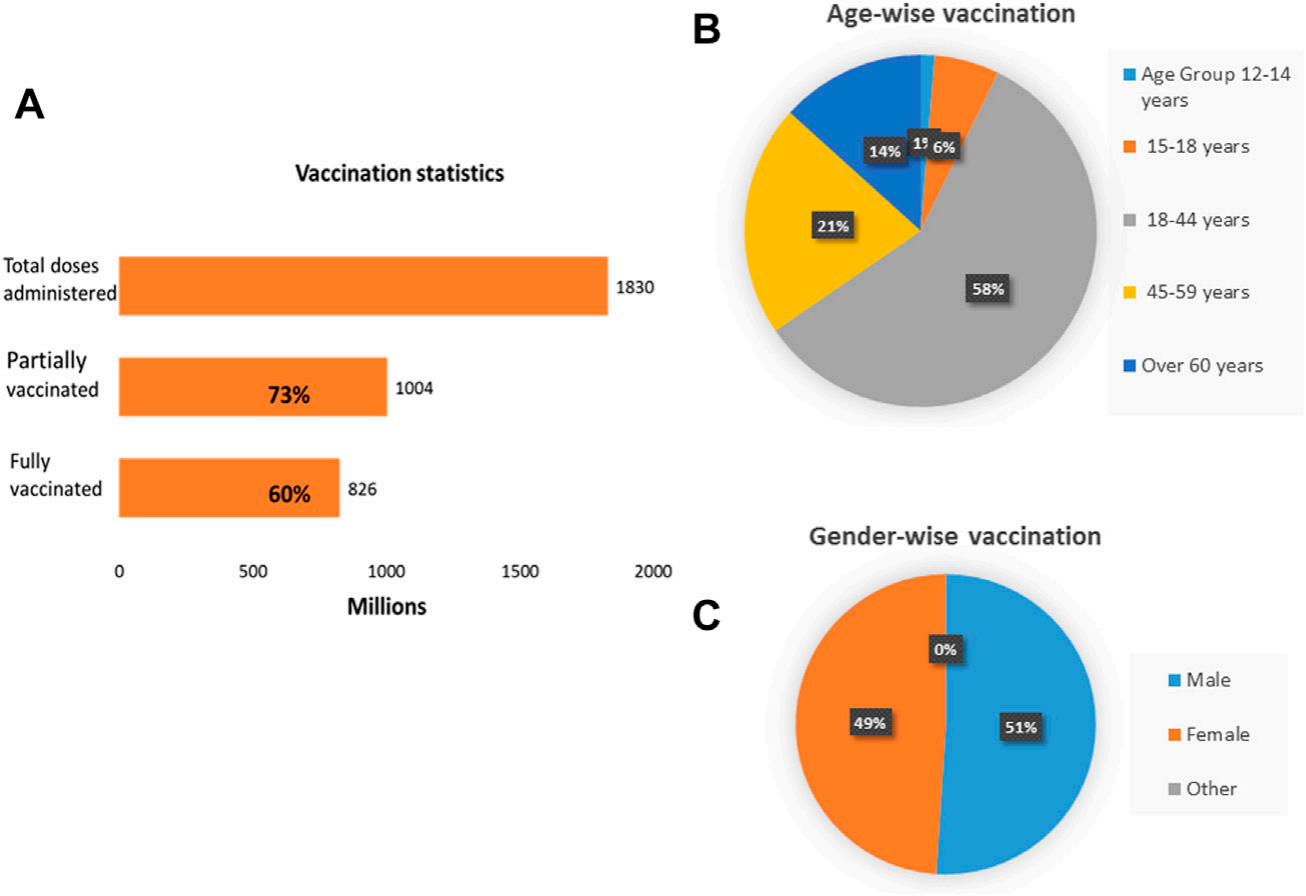
Vaccination dashboard of India. **(A)** About 1,830 million doses have been administered so far. 1,004 million individuals are partially vaccinated and 826 million individuals are fully vaccinated. (**B)** Age-wise distribution of vaccination. (**C)** Gender-wise distribution of vaccination (India, March 2022).

Mass vaccination was the main approach used by the government to ascertain immunity and protection in the general population. As of 27th March 2022, a total of 1,830,285,290 doses had been administered to eligible individuals in India. Out of it, 1,003,924,602 individuals received a single dose covering 73% of the population, while 826,360,688 were fully vaccinated representing 60% of the population [24, 32]. Table 2 depicts the list of approved vaccines in India and Figure 2 represents the vaccination status by 27th April 2022.

**TABLE 2:** List of vaccines approved and in-use in India by 27 March 2022 [36, 41, 42, 44] (India, March 2022).

Vaccine	Status	Approval	Deployment
Covishield	In use	01 January 2021	16 January 2021
Covaxin	In use	03 January 2021	16 January 2021
Sputnik V	In use	12 April 2021	14 May 2021
Moderna	Approved	29 June 2021	Order cancelled
Johnson & Johnson	Approved	7 August 2021	Deliveries expected from October 2021
ZyCoV-D	Approved	20 August 2021	Deliveries expected from October 2021
Corbevax	Approved	28 December 2021	Not yet
Covovax	Approved	28 December 2021	Not yet
Sputnik Light	Approved	6 February 2022	Not yet

Few of the government-approved and popular vaccines in India have been described as follows:

#### Covaxin

India’s first indigenous SARS-CoV-2 vaccine, Covaxin (BBV152), was developed and manufactured by Bharat Biotech (a Hyderabad-based pioneering biotechnology company) in association with the National Institute of Virology (NIV), Pune. India’s homegrown vaccine showed 64% efficacy against asymptomatic cases, 93% against severe SARS-CoV-2 infection, 78% against symptomatic cases, and 65% against the newly emerged Delta variant [33, 34]. On 2nd January 2021, BBV152 received permission for “restricted emergency use” on the recommendation of the Central Drugs Standard Control Organization (CDSCO). However, this prompt emergency approval deprived of conducting Phase III studies drew widespread criticism regarding the vaccine efficacy and safety [35]. Receiving government approval on 25th November 2020, the company started Phase III trials and by the 3rd of July 2021, vaccine efficacy data were published in medRxiv pre-print [35]. DCGI also agreed to Covaxin’s clinical trials among the children group (2–18 years) and by that time, nearly 525 volunteers were registered in the study to be conducted at AIIMS Patna and Delhi [36]. This indigenous Indian vaccine gained authorization in some other nations such as Zimbabwe, Nepal, Philippines, Iran, Guyana, Venezuela, Guatemala, and Botswana [37]. An *in vitro* preliminary study showed that the BBV152 has the potential to combat Alpha variant or lineage B.1.1.7, first identified in the United Kingdom. The vaccine had shown efficacy in neutralizing lineage B.1.617 and Zeta variant or lineage P.2 (B.1.1.28) in investigations led by ICMR and NIV, Pune respectively [38, 39]. Recently, scientists of NIV, Pune collected the sera from the already vaccinated individuals and observed substantial efficiency of Covaxin in neutralizing Beta (B.1.351) and Delta (B.1.617.2) variants [40].

#### Covishield

Covishield, trade name for Oxford–AstraZeneca COVID-19 vaccine (ChAdOx1 nCoV-19, codenamed AZD1222) was developed by Oxford University and manufactured by Serum Institute of India, Pune [41]. Covishield, the made-in-India variant of AZD1222, was the prime vaccine adopted in India’s mass immunization program and was the first vaccine to receive emergency use authorization approval by DCGI in early January 2021 [42]. A study conducted in 2020 showed that Covishield was 76% efficient in preventing symptomatic Coronavirus initially at 22 days after the first dose and 81.3% efficient following the second dose [43]. Another study performed in Scotland reported that the efficacy of AZD1222 was 81% against lineage B.1.1.7 (alpha variant), and 61% against lineage B.1.617.2 (delta variant) [44]. Following a homologous prime-boost strategy, India started a national immunization program against Coronavirus with two vaccines; Covaxin and Covishield. However, 18 persons, under the mass vaccination program, unintentionally received Covishield as the first dose and Covaxin as the second dose. ICMR institutes along with AFMC (Armed forces Medical College, Pune) compared the immunogenicity profile and safety index of these 18 individuals (heterologous group) with 40 individuals (homologous groups) who received either Covaxin or Covishield as both the doses. They observed substantially higher IgG antibody concentration and neutralizing antibody response against the delta, beta, and alpha variants in the heterologous group as compared to homologous groups of either vaccine. This study proposed that a mixed immunization scheme such as using a viral vector-based vaccine followed by a dead virus-based vaccine could not be only the safe approach but also generates better immunogenicity [45].

#### Sputnik V

Sputnik V, also known as Gam-COVID-Vac, was the world’s first registered viral vector vaccine developed by Gamaleya Research Institute of Epidemiology and Microbiology, Russia. On 12th April 2021, DCGI granted emergency use approval to Sputnik-V in India, and on 14th May 2021, the first dose was administered at Hyderabad [46, 47]. Gam-COVID-Vac comprises two genetically modified E1 gene-deficient human adenoviruses: Ad26 and Ad5 which made them replication-defective. The interim analysis of a clinical phase-III trial conducted on 19,866 Russian volunteers showed that Sputnik V has 91.6% efficacy [48]. In India, the original manufacturer joined hands with pharma giant Dr. Reddy’s Laboratories, India for the marketing and distribution of Sputnik V [49]. As of 3 August 2021, Dr. Reddy’s Laboratories received 3.15 million doses of the first component and 450,000 doses of the second component [49]. The first component of the vaccine was being produced in substantial amounts easily. However, the second component was more volatile to produce. Therefore, its availability of it challenged the overall vaccine production process. Consequently, makers of the standard vaccine developed a single-injection COVID-19 vaccine, i.e., Sputnik-Light. Sputnik-Light contains only the first component of Sputnik V (Ad26 vector) and is ideally suitable for more affected areas, permitting more individuals to be vaccinated rapidly. A real-world study in Argentina involving participants of age 60–79 years showed 79% efficiency of Sputnik-Light in preventing infection and the same results were shown in phase III clinical trial conducted in Russia [50, 51]. There were many other vaccines developed in China, the US and other countries, but we have discussed only those having a substantial share of the vaccination drive in India.

### Vaccine Acceptance and Hesitancy

COVID-19 vaccines were the most needed invention, and several research and development institutions worldwide were working on it expeditiously. However, some studies raised concerns about the acceptance of COVID-19 vaccines in terms of safety, efficacy, efficiency, negative aftereffects, vaccine necessity, misalignment with the existing health system, and insufficient awareness of vaccine-treatable illnesses among the population [52–54]. Nevertheless, vaccine hesitancy had recurrently been the foremost restraining factor in the background of the present adverse socioeconomic and health consequences. Vaccine reluctance and negligence contributed to a considerable challenge in attaining the threshold for conferring COVID-19 population-level immunity. A study on 351 highly educated (85%) participants primarily of the age group 19–29 years (>75%) was conducted to investigate the beliefs associated with the SARS-CoV-2 vaccine and obstructions leading to vaccine hesitancy among the common Indian population [55]. The vaccination acceptance rate was observed to be satisfactory, with 86.3% of participants agreeing to get vaccinated as soon as it becomes available, whereas 13.7% of participants refused vaccination entirely. However, only 65.8% of the respondents got themselves vaccinated when the COVID-19 vaccine was available to them. In this survey, 64.4% raised concern about side effects, 20.2% showed worry about vaccine efficacy and 12% thought that the SARS-CoV-2 vaccine is certainly intriguing. The need of the hour was that everyone must realize that maximum vaccination coverage was indispensable globally to curtail the pandemic. In the future, vaccine acceptance may improve through the development of trustworthy communication and information platforms to make general people aware of the benefits and availability of vaccines [56]. Further, more scientific studies on the efficacy and safety of different Coronavirus vaccines and information about their availability through centralized sources would instill confidence and resolve concerns related to vaccine reluctance. Understanding the vaccine hesitancy and the factors involved would also be useful for policymakers in planning more competent strategies for the effective implementation of mass vaccination programs.

### Impact on the Indian Economy

The pandemic had been largely disruptive to the Indian economy and could be considered as worse as the Great Depression of 1930 [57]. According to the Ministry of Statistics of India, India’s growth for the fiscal year 2020 in the fourth quarter declined as low as 3.1% [58]. Undoubtedly, India had also been experiencing economic slowdown before the pandemic with deficits in consumption as well as demand. The existing pandemic certainly has further aggravated the pre-existing threats to the outlook of the Indian economy. The World Bank reviewed the country’s growth for the financial year 2021 with the bottommost figures as compared to the same in the last three decades since economic liberalization in 1990. The gross domestic product (GDP) estimations were decreased even more to negative numbers, predicting a profound recession even after the government’s economic relief package to COVID-affected sectors in May 2020. As per the reports of the Nomura India Business Resumption Index, the Indian economic activity got down from 82.9 on 22nd March 2020 to 44.7 on 26th April 2020. During the lockdown, approximately 140 million people became unemployed while salaries came down for countless others [59, 60]. During the first complete COVID-19 lockdown (21 days) in March 2020, the economy was estimated to lose more than USD 4.2 billion every day [61] and only a quarter of the nation’s USD 2.8 trillion economic movements was effective [62].

A huge number of farmers across the nation who depend on perishable food items and daily-wage workers faced huge uncertainty in business and income. Major firms around the country such as Bharat Forge, Grasim Industries, Larsen & Toubro, Tata Motors, Aditya Birla Group, and BHEL reduced their operations or suspended temporarily while young startup businesses were also affected because their funding was stopped [63]. Indian stock markets suffered severe single (40% decline) and multi-day losses starting on March 23, 2020 [64]. Interestingly, SENSEX and NIFTY posted their largest gains on March 25th, 1 day post the 21-day lockdown announcement by the government of India [65]. On 26th March, the Indian government announced approximately USD 22 billion in economic relief funds for the poor to tackle food security, healthcare issues, and sector-related incentives. The Asian Development Bank and World Bank also sanctioned funding to India to deal with the COVID crisis [66]. The Department of Military Affairs withheld all the capital acquisitions and declared that the country will minimize the expensive defense imports [67]. The Prime Minister of India announced a USD 260 billion economic stimulus package on 12 May 2020. Signs of recovery and rebound had shown by several economic indicators by July 2020 and the nation’s economy was back to pre-COVID growth by December 2021 [68].

## Discussion

The country was able to shut down its international borders within 4 weeks since the first case of COVID-19 was identified in the country, and a nationwide complete lockdown had been imposed since 25th March 2020. India’s response to COVID-19 had been rated as one of the most rigorous across the world, surpassing Germany, Italy, the UK, the USA, and France by Oxford COVID-19 Government Response Tracker [69]. It is estimated that India would have had 0.8 million Coronavirus cases by 15th April 2020 in the absence of a timely lockdown [70]. Due to timely containment, the case number was restricted to 11,438 only as of 15th April 2020. ICMR had earlier sensed that stringent social distancing would decrease the cases by 62% [71]. Likewise, a stochastic mathematical model forecasted that continuous transmission of the virus would have led to 3 million cases by end of the May but in actuality, there were only 138,845 cases reported in India by May 25th [72].

Additionally, the government of India established approximately 600 lab facilities all over the nation, even the railway department converted 375 train coaches into COVID-19 isolation wards [73]. All possible efforts had been made to aware the public of this threat *via* social media and broadcasting. Due to the limited availability of resources in the pandemic, Indian regulatory authorities recommend that there is no necessity to affirm the results by RT-PCR or CT-scan post RAT confirmation. Immediate RT-PCR is imperative if an antigen test comes negative in an asymptomatic patient.

India managed to control the transmission of the virus in the beginning but certain demographic and economic factors made it difficult to sustain the situation. One of the major hurdles was its population density which is almost 3-times that of China. The scenario became the worst in urban slum areas where population density is more than 250,000/km^2^ making physical distancing impossible. In India, about 140 million individuals are migrants and depend on daily-wage work therefore with the sudden imposition of complete lockdown, they were forced to go back to their native places without obeying the social distancing recommended by the government [74]. Another key obstacle in India’s battle against SARS-Cov-2 had been the action and attitude of some of the residents; there had been reports of people hiding their travel history to escape from being quarantined. Some individuals took participation in forbidden massive religious meetings and became Coronavirus super-spreaders [75].

India’s poor pre-existing healthcare system is also responsible for the failure to some extent. Even though the healthcare setups had been strengthened instantly and about 2,000 dedicated COVID-19 amenities had been arranged across the nation over a very short period but the scarcity of doctors and trained nurses could not be fulfilled [76]. The country holds just 0.8 doctors/1,000 individuals in comparison to Spain’s 4.1, China’s 2.36, Iran’s 1.1, Italy’s 4.1, and the USA’s 2.6 [77]. Furthermore, Odisha and West Bengal (eastern Indian states) had recently been devastated by a super cyclone—Amphan. Homeless people were rescued from this natural calamity and placed in common shelters where physical distancing was practically impossible [78]. In the Laura Miller ranking system of healthcare, India received a “CCC” ranking and was positioned farthest among the nine participating countries suggesting major improvements in the existing healthcare system [79]. Since 2006, India spends almost 3.5% of its GDP on health and it is half of the GDP of the world expended by WHO members and by BRICS nations [80]. But the Central Bureau of Health Intelligence observed that there is just 1.28% of the government’s public expenditure (GPE) of the total revenue of the government, concluding that out-of-pocket and private health expenditures are extremely high [81]. As per National Sample Survey Office 2013–2014, out-of-pocket expenditure is a persistent concern in India as it is about 65% of the total health spending [82]. According to an OECD study, India owns 0.5 hospital beds/1,000 residents as compared to China’s 4.3 hospital beds/1,000 residents [83], indicating the need for prompt and major improvements in the public healthcare system. Another challenge faced by India during this pandemic was a short supply of goods for pharma companies. The nation used to import about 70% of the active pharmaceutical ingredients (API) for the drugs manufactured by Indian pharmaceutical companies. This huge dependency left the Indian pharma industry helpless in maintaining the supply chain as China suspended all the production facilities in the COVID-19 pandemic [84]. Consequently, this temporary shortage of API led to a hike in the price of basic medicines and supplements such as penicillin, paracetamol, and vitamins. The lesson we learned is that being one of the largest countries, India should incentivize the pharma sector to enhance the production of API which will reduce the nation’s dependency on Chinese imports and reinforce national security.

### Conclusion

India went through one of the biggest sufferings during the COVID-19 pandemic. The pandemic posed extensive health and socioeconomic challenges and also became the largest disruption in the Indian economy. In summary, we observe the major reasons behind the COVID-19 pandemic in the Indian context, to identify the pitfalls in the public healthcare system and economic preparedness to tackle a pandemic situation. We further highlight the role of citizens in aiding the fight against this kind of national or global disaster by obeying the government advisories of social distancing and containment. To minimize the dependency on other nations, India should encourage and support the in-house production of API for drug manufacturing. Fundamentally, the need of the hour for India is to put control on exponentially increasing population which is the root cause of several issues nationwide.
